# Factors associated with high vs. low implementation of Medicare’s chronic care management programme in Wyoming primary care practices

**DOI:** 10.1017/S1463423625100741

**Published:** 2026-02-12

**Authors:** Elizabeth Punke, Lucas A. Wall, Christine L. McKibbin, Catherine P. Carrico, Tonja Woods, Barbara S. Dabrowski, Abby L. Teply

**Affiliations:** 1 Department of Psychology, University of Wyoming, Laramie, WY, USA; 2 School of Pharmacy, University of Wyoming, Laramie, WY, USA

**Keywords:** Chronic care management, Evaluation, Primary care, Rural

## Abstract

**Aim:**

To identify key factors associated with varying levels of Medicare’s Chronic Care Management (CCM) programme implementation in rural primary care practices in the United States.

**Background:**

Despite demonstrated benefits for both patients and providers, CCM implementation remains low nationwide. While previous studies have examined payment-related challenges, limited research exists on other implementation factors such as leadership engagement, organizational culture, and provider training, particularly in rural settings.

**Methods:**

This mixed-methods study examined CCM implementation across six rural primary care practices in Wyoming. Thirteen healthcare professionals participated in semi-structured interviews guided by the Consolidated Framework for Implementation Research (CFIR). Practice performance data collected over three consecutive months were used to categorize sites as high or low implementers based on care coordinator productivity, percentage of care coordinated, and programme sustainability. Interview transcripts were analysed using CFIR constructs to identify factors that distinguished high from low-implementing sites, with each factor rated based on its impact (positive, negative, or neutral) and strength of influence.

**Findings:**

Three CFIR constructs strongly distinguished between high and low implementation sites: networks and communication, leadership engagement, and reflecting and evaluating. High-implementing sites demonstrated effective team communication, supportive leadership, and regular programme evaluation practices. In contrast, low-performing sites faced poor communication, minimal leadership support, and weak feedback mechanisms. Further research is needed to examine the effectiveness of targeted interventions designed to strengthen these organizational factors in rural primary care settings, particularly focusing on developing scalable strategies that account for resource limitations and geographic isolation.

The rapidly ageing population worldwide and in the United States (U.S.) faces an unprecedented burden of chronic conditions. By 2030, 1 in 6 people in the world will be aged 60 or older (World Health Organization, 2024). By this same time, the number of U.S. adults aged 65 and older will expand to 73 million – representing 1 in 5 Americans (U.S. Census Bureau, 2020). Nearly 95% of U.S. older adults have at least one chronic condition such as heart disease, cancer, or diabetes, with 80% managing multiple conditions (National Council on Aging [NCOA], 2024). These conditions not only lead the causes of death among older adults (Centers for Disease Control and Prevention [CDC], 2024a), but they also markedly impact daily functioning and independence, often necessitating institutional care or in-home support services (Hacker, 2024).

Responding to this healthcare need, the Centers for Medicare and Medicaid Services (CMS) introduced the Chronic Care Management (CCM) programme in 2015, allowing physicians, non-certified physicians, rural health clinics, and hospitals to bill for supporting care coordination of patients with two or more chronic conditions. CCM is a value-based care model with the objective to empower Medicare beneficiaries to live healthier, more independent lives through coordinated care and continuous support (Dalson, 2024). To qualify for the CCM programme, patients must have more than one chronic condition lasting 12+ months or be at risk of death, functional decline, or decompensation (when a previously stable medical condition begins to deteriorate; The Medicare Learning Network, 2022).

CCM has been shown to be beneficial for both patients and providers. For patients, CCM has demonstrated improved health behaviours, reduced medical burden, and enhanced therapy compliance and satisfaction (Yeoh *et al*., 2018). Providers and primary care practices benefit from streamlined care delivery, improved interprofessional communication and additional revenue opportunities through billable services, supporting financial sustainability (Schurrer *et al*., 2017). Despite the demonstrated benefits of CCM for both patients and providers, implementation of this programme remains low. In the first two years of the programme (2015–2016), fewer than 1% of an estimated 20 million eligible beneficiaries’ records included a CCM claim (Bindman and Cox, 2016; Schurrer *et al*., 2017). This proportion increased to just 4% by 2019 (Colligan *et al*., 2022). Low implementation represents a missed opportunity to address the growing burden of chronic diseases, improve health outcomes, and reduce healthcare costs through proactive care for older adults.

Although limited research has been conducted on the barriers and facilitators related to CCM implementation, studies by O’Malley *et al*. (2017), Bazzano *et al*. (2018) and Yeager *et al*. (2018) have each identified factors that facilitate the implementation of CCM. The authors of these studies emphasized the importance of appropriate staffing, whether through a dedicated care coordinator, such as an on-site registered nurse (O’Malley *et al*., 2017; Yeager *et al*., 2018), or through specialized staff with expertise in CCM billing and administration (Bazzano *et al*., 2018). The role of healthcare organizations was consistently emphasized, with all studies highlighting the importance of organizational support in navigating policy and implementation challenges. Additionally, the integration of CCM into electronic health records (EHR) was also an identified facilitator across studies, with customized EHR functionality or add-on modules described as critical for documenting non-face-to-face activities, tracking progress, and meeting billing requirements. Together, these findings underscore the need for a combination of dedicated personnel, technological infrastructure, and organizational guidance to support CCM adoption.

Whereas the aforementioned studies focused primarily on implementation facilitators, a more comprehensive Mathematica Policy Research Institute report on the CCM programme (Schurrer *et al*., 2017) described implementation barriers derived from interviews with 71 primary care providers and 48 CCM beneficiaries across both urban and rural settings. Interviews with providers focused on operational aspects such as billing procedures, organizational changes, and payment adequacy while interviews with beneficiaries examined experiences with consent procedures, insurance coverage, and care coordination and interviews. The authors identified several implementation barriers such as provider hesitation to recruit patients due to concerns about informed consent and cost-sharing discussions. Patients also often misunderstood programme eligibility and benefits (Shurrer *et al*., 2017). Although enrolled patients generally accepted programme requirements, particularly when they had supplemental insurance coverage (Shurrer *et al*., 2017), the study’s broad geographic scope limited its ability to examine challenges specific to rural practices. Additionally, the study did not systematically examine organizational factors that might distinguish high from low implementation, such as leadership engagement, communication systems, and quality improvement processes.

## Rural healthcare context

Rural areas may face substantial healthcare access barriers due to low population density and geographic isolation, presenting distinct challenges for healthcare delivery and for CCM (Rural Health Information Hub, 2023). Not only do rural areas experience healthcare provider and specialty provider shortages, but they also often struggle with ageing populations and higher poverty rates, which further complicate healthcare access and delivery (Rural Health Information Hub, 2023).

The Health Resources and Services Administration (HRSA) reports that approximately 75 million people live in primary care health professional shortage areas in the United States. This disparity is expected to worsen, as nonmetro areas are projected to experience a 13% increase in physician shortages compared to metro areas by 2037 (National Center for Health Workforce Analysis, 2024). Individuals in rural communities are also at greater risk of death from heart disease, cancer, unintentional injury, chronic lower respiratory disease, and stroke (Centers for Disease Control and Prevention, 2024b). The ageing of both the rural population and healthcare workforce further strains these communities. Rural areas in the United States have a higher share of older adults (65+) than urban regions, with the proportion reaching 21% in some outlying rural counties. By comparison, older adults make up 16% of the urban population and 18% of those living in suburban or micropolitan areas. Nationally, the proportion of individuals aged 65 and older is expected to rise to 24% by 2060, posing challenges for rural communities as they work to meet the needs of older residents (Warner and Zhang, 2022). Further, these demographic shifts will require a greater number of providers with specific skills and training to serve older persons (Centers for Disease Control and Prevention, 2024b).

This study was conducted in Wyoming, a predominantly rural state in the U.S. that exemplifies many of the rural healthcare challenges described above. With approximately 580,000 residents dispersed across a large geographic area (U.S. Census Bureau, 2020), Wyoming provides an ideal setting to examine CCM implementation in a rural context. Wyoming’s ageing population is increasing, with 19% of residents being over 65. Many of these older adults rely on Medicare, and an estimated 7.4% of Wyoming’s senior population lives in poverty (U.S. Census Bureau, 2020). Further, Wyoming is experiencing a significant shortage of healthcare providers, particularly in rural areas.

As of October 1, 2024, HRSA designated 40 areas within Wyoming as Primary Care Health Professional Shortage Areas, affecting approximately 182,633 residents – about 31% of Wyoming’s population (Bureau of Health Workforce, 2024). Wyoming also has a high prevalence of chronic conditions such as cardiovascular disease, diabetes, and respiratory illnesses, similar to national trends in rural areas (Bureau of Health Workforce, 2024). These healthcare delivery challenges, combined with an ageing Medicare-eligible population, create a compelling need for coordinated care management.

## Research questions

1. What factors are associated with high versus low implementation of the CCM programme in rural primary care settings?
2. What specific facilitators and barriers influence CCM implementation, and what challenges do healthcare providers face in engaging with and sustaining CCM services?

## Conceptual framework

The Consolidated Framework for Implementation Research (CFIR, 2009) served as a conceptual foundation guiding this study. The CFIR is a practical, theory-driven model designed to systematically assess barriers and facilitators to implementation (Damschroder *et al*., 2009). CFIR constructs provide a lens for identifying factors that influence the adoption and success of interventions, offering guidance on tailoring strategies, making necessary adaptations, and explaining outcomes (Riffin *et al*., 2024). Recognized for its ability to capture diverse implementation factors across settings and interventions (Damschroder *et al*., 2009; Kirk *et al*., 2015), the CFIR was selected for this study due to its comprehensiveness and its capacity to reflect the nuanced barriers and facilitators specific to CCM implementation (Varsi *et al*., 2015).

The CFIR model is composed of five primary domains – intervention characteristics, outer setting, inner setting, characteristics of the individuals involved, and the process of implementation – encompassing 39 constructs (CFIR, 2009). The first CFIR domain, *intervention characteristics*, focuses on the features of the intervention being implemented. Interventions, if not adapted, can be a poor fit for the organization, leading to resistance from those affected (Pettigrew *et al*., 2001). The next two domains, *inner setting* and *outer setting*, relate to the organizational and external contexts in which implementation occurs. The outer setting includes factors like economic, political, and social conditions, while the inner setting refers to the organization’s structural, political, and cultural features (Pettigrew *et al*., 2001). The fourth domain, *characteristics of individuals*, addresses the role of people involved in the intervention and implementation process. Characteristics of individuals are important because individuals have agency and can influence the outcome of implementation through their behaviours, mindsets, and professional affiliations (Pettigrew *et al*., 2001). The final domain, the *implementation process*, encompasses a series of actions required to implement the intervention. This process is typically non-linear, involving a combination of planned and spontaneous activities, including reflection and assessment. Individuals from both the inner and outer settings may play key roles as change agents, promoting the intervention’s success through various strategies (Pettigrew *et al*., 2001). Effective implementation depends on aligning these processes at multiple levels within the organization (Pettigrew *et al*., 2001). Therefore, this study aims to address these gaps by examining factors associated with high and low CCM implementation across rural primary care practices, using Wyoming as our study setting.

## Method

### Study design

This study was conducted using a convergent mixed-methods approach (Creswell and Creswell, 2018) to integrate quantitative implementation metrics with qualitative provider insights to identify key factors influencing the implementation of the CCM primary care practices in Wyoming. A convergent mixed-methods approach was selected for this study because this design allows for simultaneous collection of quantitative data (implementation metrics) and qualitative data (provider experiences through interviews), with integration of findings providing a more comprehensive understanding of implementation factors than either method alone.

### Participants

Participants were healthcare professionals working in primary care practices that had implemented Medicare’s CCM programme in Wyoming. Eligible participants met three criteria: (1) employment at a CCM-implementing primary care practice, (2) direct involvement in CCM delivery, and (3) affiliation with the Wyoming Centre on Ageing and use of the CrossTx (https://www.crosstx.com/) platform for tracking service delivery. CrossTx is a care coordination platform that optimizes patient outcomes through efficient workflows and data integration while supporting care continuity and compliance with healthcare standards.

### Procedure

Qualitative interviews were conducted by the trained study coordinator and were designed based on the CFIR model to explore participants’ experiences with CCM implementation. Table 1 outlines the five CFIR domains, provides descriptions of each domain, and presents sample interview questions used in this study. Interviews were conducted via Zoom, lasting 50–60 minutes. The interview guide included one overarching, open-ended question for each domain, supplemented by sub-question prompts that were consistent with the CFIR interview guide addressing specific constructs, such as adaptability, patient needs, networks and communication, and knowledge and beliefs about the intervention. This flexible structure allowed for in-depth exploration while ensuring all relevant CFIR domains and constructs were addressed. Interviews were audio-recorded, transcribed, and de-identified. Concurrently, practice-level performance data were collected over three consecutive months using data derived from CrossTx software reports to categorize sites as either high or low CCM implementers. Because the primary goal of this project was quality improvement, the University of Wyoming Institutional Review Board classified the project as ‘not research.’ Despite this exempt status, the quality improvement team obtained written informed consent from all participants prior to their involvement in the study. Verbal consent was also obtained and recorded at the beginning of each interview session.

**Table 1.** CFIR domains, descriptions, and sample interview questions used in the study

CFIR domain	Description	Sample interview questions
Intervention characteristics	Attributes of the intervention that influence implementation success, including its complexity, adaptability, quality, and perceived advantage over existing practices.	• Why was CCM implemented? • How was the decision made to implement it? • How complicated is CCM to implement?
Outer setting	External influences including patient needs, health policy, reimbursement structures, and relationships with other healthcare organizations.	• How well do you think CCM has met the needs of older adults served in your practice? • What kind of financial or other incentives have influenced your degree of success with CCM?
Inner setting	Internal organizational features affecting implementation, including culture, communication networks, leadership engagement, and available resources.	• What kinds of infrastructure changes were needed to accommodate the intervention? • Tell me about a time you needed to work with colleagues to implement this intervention.
Characteristics of individuals	Knowledge, beliefs, self-efficacy, and other attributes of the people involved in implementation.	• How do you feel about the intervention being used in your setting? • How confident were you that it could be implemented successfully here?
Process	Activities undertaken to implement the intervention, including planning, engaging stakeholders, executing changes, and evaluating progress.	• Who was involved with the planning process? What were their roles? • To what extent did your organization set goals around CCM?

### Quantitative measures

Sites were classified using rural-urban commuting area (RUCA) codes from the USDA Economic Research Service’s 2010 ZIP code database. RUCA codes range from 1.0 (metropolitan) to 10.0 (isolated rural), with codes above 4.0 indicating rural areas. Three implementation metrics were collected from CrossTx reports: (1) Care coordinator productivity, was measured by comparing each participant’s actual caseload to the expected caseload based on their full-time equivalent (FTE) value. The benchmark threshold was defined as managing at least 80% of the expected caseload; (2) Billing efficiency was assessed by comparing the number of patients for whom a bill was generated to the total number of patients enrolled in CCM in a given month. A percentage was calculated and a threshold was defined as billing for 80% or more of enrolled patients over three months; and (3) Programme sustainability was calculated using practice net revenue over three months, adjusted for Wyoming’s average registered nurse hourly wage and cost of living ($41.17; Robertson and Falvey, 2023). The threshold for sustainability was defined as generating sufficient revenue to cover at least 80% of the care coordinator’s salary and benefits. Based on these metrics collected over three consecutive months, practices exceeding thresholds in two or more areas were categorized as ‘high’ implementation sites; the remainder were classified as ‘low’ implementation sites.

### Survey measures

A demographics and clinical training survey collected comprehensive information about participants’ professional backgrounds, including their health profession, specific roles within their practice, professional credentials, length of time at their current practice, experience with CCM programme implementation, responsibilities for patient enrolment, and characteristics of their patient panels.

### Recruitment

Recruitment occurred at both practice and individual levels using purposive sampling. At the practice level, invitational letters were sent to primary care practice managers in Wyoming who had implemented the CCM programme as part of a larger quality improvement initiative. These letters were followed by phone calls to encourage participation. Once practice managers agreed to participate, quality improvement staff scheduled a 10-minute presentation during a staff meeting to introduce the project and invite staff to participate in a semi-structured interview. Interested staff members were invited to indicate interest either immediately by placing their information on a sign-up form or later via a Qualtrics link. The Qualtrics link led to a page where participants could access detailed project information, an informed consent document, and a demographics and clinical training survey to collect information about participants’ professional backgrounds such as their health profession, role(s), credentials, practice tenure, CCM experience, enrolment responsibilities, and patient panel characteristics.

### Data analysis

Qualitative data from interviews were transcribed verbatim and analysed using a deductive approach based on the CFIR model. Three investigators coded the data using definitions from the five domains of the CFIR and 39 constructs (CFIR, 2009). The investigators, following methods adapted from Varsi *et al*., (2015), assigned numerical ratings to reflect both the valence (positive/negative influence) and strength of impact on CCM implementation. These valence ratings ranged from −2 (strong negative) to +2 (strong positive). Following completed ratings, a matrix was created to organize the CFIR constructs and their ratings, comparing high and low implementation sites to identify distinguishing patterns.

Whereas Varsi *et al*., (2015) required two staff from each site to endorse a strongly positive or negative valence to generate a score of 2, this study required one staff person to endorse a strong valence. This adaptation was made due to small sample size. Not all sites had more than one staff person who participated. Constructs were classified as ‘strongly distinguishing’ when high implementation sites demonstrated consistently positive influence (rated 0 to +2, with at least one site rated +2) while low implementation sites showed consistently negative influence (with at least one site rated −2). Constructs were classified as ‘weakly distinguishing’ when high implementation sites showed neutral to mildly positive influence (rated 0 to +1) while low implementation sites showed at least one instance of negative influence (rated −1).

Quantitative data were analysed using descriptive statistics in IBM SPSS Statistics (Version 28.0). Practice performance was evaluated by deriving the metrics for care coordinator productivity, percentage care coordinated, and programme sustainability from CrossTx reports averaged over three consecutive months. Practices meeting two or more criteria were categorized as high implementation sites; the remainder were classified as low implementation sites.

## Results

Thirteen healthcare professionals from six rural Wyoming primary care centres participated in the study. According to the RUCA Codes developed by the U.S. Department of Agriculture (2022), sites were distributed across metropolitan (RUCA 1.0, *n* = 1), micropolitan (RUCA 4.0, *n* = 1), small rural towns (RUCA 7.0, *n* = 3), and isolated rural areas (RUCA 10.0, *n* = 1). The majority of participants were non-Hispanic, White, cisgender women (92%, *n* = 12). The most common professional roles were nursing (46%, *n* = 6) and pharmacy (23%, *n* = 3), and several held multiple roles (46%, *n* = 6), including nurse, care coordinator, and pharmacist. The experience of participants with the CCM programme ranged from <6 months to >5 years. Approximately one half of the participants (54%, *n* = 7) were responsible for enrolling patients in the CCM programme.

CCM implementation varied among the six primary care practices studied. As noted in Table 2, Care Coordinator Productivity ranged from 15% to 137% of expected caseloads (based on FTE values of 0.2 to 1.0). Medicare beneficiary enrolment spanned from 19 to 127 patients per practice, with total enrolment across sites increasing from 360 to 382 during the study period (Table 3). Monthly billing ranged from $417 to $18,588, with net revenue varying from −$3,151 to $11,451. Table 4 displays the primary care centre billing and net revenue accounting for average Wyoming RN labour costs ($41.17 per hour; Robertson and Falvey, 2023).

**Table 2.** Care coordinator’s productivity as determined by their position’s FTE value

	Mean	SD	Range
Month prior to qualitative interview	88.2%	15.8%	50–100%
Month of qualitative interview	97.0%	44.0%	25–147%
Month following qualitative interview	78.5%	35.8%	27–137%

**Table 3.** Medicare beneficiaries in CCM enrolment and billable services

Number of medicare beneficiaries enrolled in CCM			
	Mean	SD	Range
Month prior to	60	33.59	19–104
Month of	64	37.70	19–120
Month following	63	42.58	19–127
Number of Medicare Beneficiaries Receiving Billable Service for CCM			
	Mean	SD	Range
Month prior to	68	32.1	19–98
Month of	57	31.5	19–95
Month following	60	39.9	5–123
Proportion of Enrolled Beneficiaries Receiving Billable Services for CCM			
	Mean	SD	Range
Month Prior to	96.7%	3.7%	92%–100%
Month of	93.3%	7.2%	79%–100%
Month following	88.2%	15.8%	50%–100%

**Table 4.** Primary care centre revenue and net revenue after accounting for average Wyoming RN labour costs

Primary care centre’s revenue			
	Mean	SD	Range
Month prior to	$6713.31	$4460.05	$1546.00–$15,692.78
Month of	$6885.182	$5792.845	$1546.00–$18,588.00
Month following	$6756.73	$5570.58	$417.00–$17,201.96
Primary care centre’s net revenue inclusive of Wyoming labour costs for a registered nurse position			
	Mean	SD	Range
Month prior to	$3133.18	$2703.62	−$1823.24–$8556.65
Month of	$3138.71	$4367.15	−$1706.27–$11,451.87
Month following	$3010.27	$3810.66	−$3151.07–$10,065.83

Based on the three success criteria, as shown in Table 5, (Care Coordinator Productivity, Percentage Care Coordinated, and Programme Sustainability), four sites were classified as high implementation and two as low implementation.

Qualitative data were analysed for strength and consistency across high implementation sites and low implementation sites. Results of this analysis are presented in Table 6. Analysis through the CFIR framework revealed both strongly and weakly distinguishing constructs between high and low implementation sites. Networks and Communication, Leadership Engagement, and Reflecting and Evaluating emerged as strongly distinguishing constructs, while Relative Advantage and Implementation Climate were identified as weakly distinguishing constructs.

**Table 5.** Classification of primary care practice sites as ‘high’ or ‘low’ implementation according to three metrics

Practice	Care coordinator productivity	Billing efficiency	Programme sustainability	Classification as high or low implementation
1	Productive	Efficient	Sustainable	High
2	Unproductive	Efficient	Sustainable	High
3	Productive	Efficient	Sustainable	High
4	Productive	Efficient	Sustainable	High
5	Unproductive	Efficient	Unsustainable	Low
6	Unproductive	Efficient	Unsustainable	Low

**Table 6.** Matrix of valence ratings of CFIR constructs per primary care centre

Ratings assigned to CFIR constructs	High implementation sites				Low implementation sites	
Site ID	1	2	3	4	5	6
I. Intervention Characteristics						
Intervention Source	–	–	–	–	–	–
Evidence Strength and Quality	(+2)	(+2)	–	–	–	–
Relative Advantage*	(+2)	(+2)	(+2)	(+1)	(−1)	(+2)
Adaptability	–	(+1)	–	(−2)	(−2)	–
Trialability	–	–	–	–	–	–
Complexity	0	(+1)	(+1)	0	–	(+1)
Design Quality and Packaging	(−2)	(+1)	(−2)	–	(−2)	–
Cost	0	(−1)	(+1)	(−2)	–	(+1)
II. Outer Setting						
Patient Needs and Resources	0	(+2)	(+1)	(+2)	(+1)	(+2)
Cosmopolitanism	(+2)	(+2)	(+2)	(−2)	–	(+2)
Peer Pressure	–	–	(+1)	–	–	–
External Policies and Incentives	–	(−2)	(+2)	(−2)	–	(−1)
III. Inner Setting						
Structural Characteristics	0	(+2)	0	(−1)	–	0
Networks and Communication**	(+2)	(+1)	(+2)	(+1)	(−2)	(+2)
Culture	(−1)	(+2)	0	(+2)	(−1)	(+2)
Implementation Climate*	(+1)	(+1)	0	(+1)	(−1)	(+1)
Tension for Change	–	(+1)	–	–	–	–
Compatibility	(+1)	(+2)	(−1)	(+1)	–	(+2)
Relative Priority	(+1)	(+2)	(−2)	(−2)	(−2)	(−1)
Organizational Incentives and Rewards	–	–	(+1)	–	–	0
Goals and Feedback	(+2)	(+2)	(−2)	(−1)	(−2)	(−1)
Learning Climate	(+1)	(+2)	(+1)	–	–	–
Readiness for Implementation	–	(+1)	–	–	–	–
Leadership Engagement**	0	(+2)	0	–	(−1)	(−2)
Available Resources	(+1)	(+1)	0	(−2)	(−2)	(+2)
Access to Information and Knowledge	(−2)	–	(−2)	–	–	(+1)
IV. Characteristics of Individuals						
Knowledge and Beliefs about the Intervention	(+2)	(+1)	(+2)	–	(+1)	(+1)
Self−efficacy	(+1)	0	0	(+1)	(+2)	(+1)
Individual Stage of Change	–	0	(+1)	(+2)	–	–
Individual Identification with Organization	–	–	–	–	–	–
Other Personal Attributes	(+2)	(+1)	(+2)	(+1)	(+1)	(+1)
V. Process						
Planning	(+1)	(+2)	(−1)	(+1)	–	–
Engaging	(+2)	(+1)	0	(−1)	–	–
Opinion Leaders	0	(+2)	–	–	–	–
Formally Appointed Internal Implementation Leaders	(+2)	(+2)	(+2)	0	0	0
Champions	(+2)	–	(+2)	(+1)	(+2)	(+1)
External Change Agents	(+2)	(+2)	(+2)	(+2)	(+2)	(+2)
Executing	(+2)	(+2)	(+1)	(+1)	(+2)	–
Reflecting and Evaluating**	(+1)	(+2)	(+1)	(+2)	(−2)	–

** Construct strongly distinguishes between low and high implementation effectiveness.

* Construct weakly distinguishes between low and high implementation effectiveness.

*Note*: - indicates data not reported.

### Strongly distinguishing constructs

Networks and Communication – defined as how well people connect and interact within an organization, both through official channels and informal relationships (CFIR, 2009) – strongly differentiated high from low implementation sites. At high implementation sites, interviewees consistently highlighted frequent, efficient, and collaborative communication practices within the organization that supported enhanced patient care. One participant described how regular team meetings and clear communication with leadership fostered efficient care coordination, ‘*The CCM Team meets on a weekly basis just to talk about CCM patients… our collaboration is on par*’ (Participant 4, Practice 2). Conversely, interviewees at low implementation sites reported frustration with delayed and inadequate communication, noting a lack of regular meetings and unclear dissemination of information. One participant emphasized the need for better communication, stating, ‘*They don’t disseminate that information to the ones that are doing the work*’ (Participant 8, Practice 5).

Leadership Engagement – defined within the Inner Setting domain as how actively involved, committed, and accountable leaders are in implementing changes (CFIR, 2009) –strongly differentiated between high and low implementation sites under the Inner Setting domain. At high implementation sites, interviewees consistently praised their leaders for being receptive, committed, and supportive of the CCM programme, which they felt enhanced its success. For example, one participant emphasized ‘*100% support*’ from leadership (Participant 4; Practice 2). In contrast, interviewees from low implementation sites described their leaders as unreceptive, lacking support and understanding of CCM, and failing to provide feedback on the programme. One participant from a low implementation site expressed frustration with their leader’s lack of support, stating, ‘*I haven’t heard a lot…. So, I don’t know what to say*’ (Participant 11, Practice 6). Challenges such as staffing shortages, integration issues with existing EHR systems, and negative cultural aspects were identified as barriers to effective implementation.

Reflecting and Evaluating – defined as how data and feedback are used to track implementation progress, combined with regular team discussions about experiences and outcomes (CFIR, 2009) – was the only construct in the Process domain that strongly distinguished between high and low implementation sites. High implementation sites actively used quantitative and qualitative data to evaluate programme success, reporting positive experiences with reflection and assessment. Interviewees also noted that staff receptiveness to feedback from colleagues and leaders was beneficial for programme implementation. For example, a provider at a high implementation site reported, ‘*hard data to prove that we’re improving patients’ health’* (Participant 4, Practice 2) and improved haemoglobin A1C levels as indicators of programme effectiveness. In contrast, interviewees from low implementation sites noted a lack of feedback or evaluation, with one participant expressing feeling unrecognized and unsupported in their efforts, ‘*I’ve not been told if I’ve been doing a good job or a bad job*’ (Participant 8, Practice 5). This absence of reflection and evaluation hindered progress and left staff feeling disengaged.

### Weakly distinguishing constructs

Relative Advantage – defined as how much better stakeholders believe implementing a new solution would be compared to other options (CFIR, 2009) – was the only construct in the Intervention Characteristics domain that weakly distinguished between high and low implementation sites. Interviewees from high implementation sites expressed positive views on the relative advantages of CCM, citing benefits such as improved communication with patients, better care coordination, and enhanced patient outcomes. For example, a participant from a high implementation site noted, *‘I think the CCM program is a fantastic opportunity to enhance the care of geriatric patients. I think it provides them with an opportunity to communicate more with their provider… to communicate more with healthcare workers in general’* (Participant 3, Practice 1). Conversely, a participant from a low implementation site described their view of CCM as offering no additional health support compared to standard care, *‘It doesn’t offer anything extra. There’s no additional health support or follow-ups compared to what we were already doing’* (Participant 8, Practice 5). This construct was categorized as weakly distinguishing rather than strongly distinguishing because, while differences existed between high and low implementation sites, they did not meet the threshold for strong distinction (i.e. high sites rated +2 and low sites rated −2). Instead, high implementation sites generally showed moderately positive perceptions (rated +1) while low implementation sites showed mild negative perceptions (rated −1).

Implementation Climate – defined as an organization’s readiness and ability to adopt changes, including how receptive people are to new initiatives and whether they will be supported in using them (CFIR, 2009) – was the second construct in the Inner Setting domain that weakly distinguished between high and low implementation sites. At high implementation sites, while some resistance was initially reported, it often diminished as the benefits of CCM became more evident, with some sites noting no resistance at all. As one participant stated, *‘Everyone in the organization supports [CCM]. And it’s really become more of a team-based, organization-wide effort versus just a clinic effort. And we’re continuing to get that buy-in from everyone’* (Participant 13, Practice 3). Another high implementation participant noted that staff receptivity to CCM was influenced by workload and their ability to manage the programme independently, stating *‘[CCM] works best not to depend on anybody else. That’s what I know… I mean as long as I stayed and don’t expect anything from somebody else, [CCM] fit really well’* (Participant 7, Practice 3). At low implementation sites, while staff weren’t outwardly resistant, their acceptance appeared more passive. As one participant noted, “*Everybody was okay with it, provider-wise. Our manager at the time just said, ‘this is something we’re gonna do’*” (Participant 11, Practice 6). This less enthusiastic reception was described as contributing to ongoing implementation challenges.

## Discussion

The aim of this study was to identify key organizational factors influencing CCM implementation across six rural primary care practices in Wyoming. Guided by the CFIR (2009) framework, we identified three strongly distinguishing constructs (networks and communication, leadership engagement, and reflecting and evaluating) and two weakly distinguishing constructs (relative advantage and implementation climate) between high and low-implementing sites. The study took place in rural Wyoming – a unique context for examining the implementation of CCM.

Rural populations are disadvantaged and, in many cases, health care systems fail to reach these populations at same levels as for urban populations. Worldwide, access to health care services is lower for people living in rural than urban areas (WHO, 2018). Individuals living in rural communities in the U.S., including Wyoming, experience higher rates of disabilities (Zhao *et al*., 2019), lower utilization of preventive services (Schroeder, 2018), and lower health status, with 11.2% reporting ‘fair/poor’ health compared to 8.4% in urban areas (National Center for Health Statistics [NCHS], 2018). These disparities are further compounded by disadvantaged socioeconomic factors (e.g. lower income levels, limited insurance coverage) and limited access to healthcare resources (e.g., fewer specialists, greater travel distances to facilities; Douthit *et al*., 2015). These challenges likely intensify barriers to CCM implementation, making the findings particularly relevant for understanding factors affecting CCM uptake and sustainability in resource-limited rural primary care settings.

The emergence of networks and communication as a strongly distinguishing construct highlights unique challenges in rural healthcare delivery. While high-implementing sites established formal communication protocols and regular team meetings, low-implementing sites struggled with information dissemination and care coordination. This finding reveals a distinction from previous CCM research, as formal communication structures appear particularly essential in rural settings, as compared to urban settings where informal communication channels may be more readily available and sufficient (Schurrer *et al*., 2017). In rural Wyoming, where practices often operate with minimal staffing and across vast geographic distances, intentional communication structures become critical for programme success. High-implementing sites overcame these challenges through structured weekly CCM team meetings and clear protocols for information sharing. This finding aligns with broader implementation science literature, as Damschroder and Lowrey (2013) emphasized the role of team relationships in implementation success. Our findings also complement the Mathematica Policy Research report (Shurrer *et al*., 2017) which identified communication as crucial for patient engagement in CCM programmes. Our study specifically highlights its importance for organizational functioning in resource-constrained settings.

Leadership engagement also emerged as a critical distinguishing factor, with high-implementing sites characterized by proactive and supportive leadership. Effective leaders at high-implementing sites were reported to have strong communication skills and adaptability– qualities identified as essential for rural healthcare leadership (National Rural Health Association [NRHA], 2024). In rural settings, healthcare administrators often oversee multiple facilities and operate under constrained budgets, which can impede CCM implementation. Strong leadership in these settings can help mitigate resource shortages by fostering staff engagement, securing external funding, and advocating for policy adaptations to accommodate rural healthcare challenges (Varsi *et al*., 2015). Our quantitative data support this relationship; sites with strong leadership engagement showed high coordinator productivity and adequate revenue generation.

The importance of reflecting and evaluating in distinguishing implementation success reveals how rural practices can overcome data infrastructure limitations. High-implementing sites developed creative approaches to programme evaluation, combining quantitative metrics with qualitative feedback despite limited access to sophisticated data systems. This adaptability was important for maintaining programme growth and momentum and demonstrating value to stakeholders, with high-implementing sites reporting concrete improvements in patient outcomes (e.g. improved haemoglobin A1c levels) despite rural resource constraints. This finding is consistent with Damschroder and Lowrey’s (2013) study, which found that reflecting and evaluating was one of the key constructs distinguishing between low and high implementation effectiveness. In the context of rural Wyoming, where access to centralized data systems may be limited, high implementation sites found alternative methods to track progress, such as leveraging informal feedback loops and adapting evaluation processes to fit local constraints. This highlights the importance of flexible, context-specific evaluation strategies for sustaining programmes like CCM in rural settings.

The weakly distinguishing constructs – relative advantage and implementation climate – highlight how staff perceptions influence CCM success in rural settings. Sites where staff recognized CCM’s benefits despite implementation challenges showed improved programme sustainability and patient engagement, suggesting that rural practices must actively build buy-in while acknowledging contextual constraints. This finding extends Damschroder and Lowery’s (2013) work by demonstrating how perception of intervention value interacts with rural-specific barriers like workforce shortages and technology limitations. In our study, sites with positive implementation climates developed workarounds for resource limitations, such as cross-training existing staff and adapting workflows to accommodate constraints. As noted by Damschroder and Lowery (2013), organizational readiness and climate for implementation can help overcome structural barriers, which appears particularly relevant in resource-constrained rural settings.

The CFIR framework has been applied across various healthcare contexts to evaluate barriers and facilitators to the implementation of healthcare interventions, and the inner setting domain has been consistently identified as a critical domain influencing implementation success (Damschroder and Lowrey, 2013; Kramer *et al*., 2017; Pratt *et al*., 2022). Despite the differences in settings and interventions, the consistency in the importance of the inner setting domain in shaping implementation success underscores the fundamental importance of these factors in shaping the success of CCM implementation across diverse primary care environments. However, this study suggests that while these factors are important across a variety of settings, their role in rural healthcare may be even more pronounced. The scarcity of specialized staff, limited infrastructure, and geographic isolation in rural Wyoming heighten the importance of strong communication, leadership, and adaptive evaluation to overcome implementation challenges.

## Implications

These findings have several implications for practice and policy. Literature indicates that healthcare organizations implementing programmes like CCM may benefit from prioritizing three key areas: communication networks, leadership engagement, and evaluation practices (Kirk *et al*., 2015). In rural contexts, achieving these goals could require specific adaptations. For example, organizations might consider leveraging telehealth platforms for interdisciplinary collaboration where feasible, though implementation success likely depends on local broadband infrastructure and staff technology comfort levels (Douthit *et al*., 2015). Evidence also suggests that providing targeted leadership training to clinicians managing multiple roles may help address staffing constraints that are commonly experienced in rural settings (NRHA, 2024).

In building positive implementation climates for programme sustainability, organizations could consider several evidence-based strategies (Damschroder and Lowery, 2013; O’Malley *et al*., 2017). These include empowering local staff through shared decision-making processes; establishing mentorship programmes between experienced and new CCM providers; committing to high quality, structured, continuous communication between frontline staff and leadership; incentivizing engaged staff; and developing context-specific training that addresses rural healthcare challenges. It maybe be particularly important for leadership to identify, engage, and reinforce team members who take responsibility for programme implementation (e.g. champions). These team members can engage others in continuous quality improvement and can provide critical support for programme continuity through timely training of new staff in the face of turnover. Leadership engagement and reinforcement of and direct communication with these individuals is particularly important to maintain momentum for and sustainability of implementation efforts.

Rural clinics may particularly benefit from tailoring their approach to acknowledge resource limitations while emphasizing CCM’s potential benefits. Versatile care coordinator roles may be seen in these settings to maximize productivity, due to staffing constraints. In addition, integration of students from rural community colleges and universities (e.g. social work, pharmacy, and nursing programmes) may also help to extend the rural workforce in their implementation efforts. Importantly, research shows that effective implementation requires balancing workload with demonstrated patient outcome improvements (O’Malley *et al*., 2017). High-implementing sites often track and share metrics that are meaningful to rural providers and organizational leadership, such as reduced emergency department visits or improved chronic disease management among geographically isolated patients. Therefore, a focus on tracking meaningful process measures, outcomes, and metrics may be important to maintain engagement at all levels from frontline staff to those in leadership.

## Limitations

This study is not without its limitations. The small sample size (six practices) and focus on Wyoming may limit generalizability to other rural contexts. It is also possible that practices who elected to participate were more likely to engage in quality improvement activities due to their self-reflection, which may have biased the results toward more favourable implementation characteristics. With respect to methods used, the adaptation of the Varsi *et al*. (2015) rating method to require only one staff member’s endorsement for a strong rating (+2 or −2) may have resulted in a construct being classified as ‘strongly distinguishing’ based on a single, particularly emphatic opinion, which is an important consideration given the small sample size. Additionally, while the 2009 CFIR framework provided structured approach, its broad scope and interconnected constructs can complicate coding and interpretation. Limited information was provided by participants relating to the process domain, specifically the reflecting and evaluating construct of implementation. (Damschroder and Lowrey, 2013; Varsi *et al*., 2015). Future research should examine how these findings translate across different rural contexts and explore how CFIR might be adapted for rural healthcare settings.

## Conclusion

This study advances understanding of CCM implementation in rural primary care by identifying key organizational factors that differentiate high and low-implementing practices.

The findings highlight how rural context shapes implementation dynamics and reinforces the need for rural-specific implementation strategies that acknowledge the distinct resource constraints and logistical challenges of delivering CCM in underserved areas. Future research should explore how rural practices can leverage existing strengths to enhance CCM adoption and sustainability while addressing unique geographical and resource constraints.

## Data Availability

Data are available upon reasonable request from the corresponding author.
